# Social coordination in animal vocal interactions. Is there any evidence of turn-taking? The starling as an animal model

**DOI:** 10.3389/fpsyg.2015.01416

**Published:** 2015-09-28

**Authors:** Laurence Henry, Adrian J. F. K. Craig, Alban Lemasson, Martine Hausberger

**Affiliations:** ^1^Laboratoire d'éthologie animale et humaine, Centre National de la Recherche Scientifique, UMR 6552, Université de Rennes 1Rennes, France; ^2^Department of Zoology and Entomology, Rhodes UniversityGrahamstown, South Africa; ^3^Laboratoire d'éthologie animale et humaine, Centre National de la Recherche Scientifique, UMR 6552, Station Biologique, Université de Rennes 1Paimpont, France

**Keywords:** turn-taking, vocal interactions, conversation rules, mammals, birdsong, sturnids

## Abstract

Turn-taking in conversation appears to be a common feature in various human cultures and this universality raises questions about its biological basis and evolutionary trajectory. Functional convergence is a widespread phenomenon in evolution, revealing sometimes striking functional similarities between very distant species even though the mechanisms involved may be different. Studies on mammals (including non-human primates) and bird species with different levels of social coordination reveal that temporal and structural regularities in vocal interactions may depend on the species' social structure. Here we test the hypothesis that turn-taking and associated rules of conversations may be an adaptive response to the requirements of social life, by testing the applicability of turn-taking rules to an animal model, the European starling. Birdsong has for many decades been considered as one of the best models of human language and starling songs have been well described in terms of vocal production and perception. Starlings do have vocal interactions where alternating patterns predominate. Observational and experimental data on vocal interactions reveal that (1) there are indeed clear temporal and structural regularities, (2) the temporal and structural patterning is influenced by the immediate social context, the general social situation, the individual history, and the internal state of the emitter. Comparison of phylogenetically close species of Sturnids reveals that the alternating pattern of vocal interactions varies greatly according to the species' social structure, suggesting that interactional regularities may have evolved together with social systems. These findings lead to solid bases of discussion on the evolution of communication rules in relation to social evolution. They will be discussed also in terms of processes, at the light of recent neurobiological findings.

## Introduction

### The human “bases”

Vocal communication is widespread in the animal kingdom and vocal interactions are an important part of social functioning. Temporal and structural regularities depend on the species' social structure, or may even depend on the immediate context. Two extremes are generally encountered, with either an overlap superposition of acoustic signals between interlocutors or a strict alternation of vocal utterances: a first emitter leaves a silent interval before producing the following sound during which the second emitter can respond. As in humans, animal vocal interactions may be dyadic (“face to face”) or at the group level.

The question though is to what extent these regularities may be functionally convergent with human communication rules, such as turn-taking.

According to Logue and Stivers (2012), the analysis of conversation in humans is based on methods and theories that emerged from sociology in the 70s. One “founder” paper was that of Sacks et al. (1974) who formalized the basics of turn-taking rules and defined them according to eight characteristics (see also Craig and Washington (1986): (1) only one person speaks at a time, (2) the number of participants may vary, (3) the order for speaker turns is variable, (4) turn size is not fixed, (5) the content of speaker turns is spontaneous, (6) simultaneous speech is infrequent and brief, (7) techniques exist for repairing turn exchange errors, (8) turn allocation techniques are used to regulate the exchange. The two major elements are the alternation of utterances between interlocutors and the avoidance of overlap, hence temporal features. In most human cultures, overlap appears as a conversation failure (Sacks et al., 1974) and can lead to the end of the exchange. In human conversation, the fundamental frequency declines, changes in gazing and other subtle signs are used to guide conversational turn-taking (Gérard, 1987; Hauser, 1992). Another important point is that conversational turn-taking rules are acquired during development through adult modeling (Locke, 1993). It is even considered as a child's major achievement, which is made possible by the early stages of parent-child interaction (e.g., Rutter and Durkin, 1987). The mother is seen by some authors as controlling the child rather than facilitating it in the mother-child dyadic interactions (Miura, 1993). Adults may play a major role in canalizing the flow of speech so that it is fragmented enough to allow turns between speakers. Neglected children fail to develop this ability, showing irrelevant turns, interruptions, simultaneous talking and non-contingent responding (Black and Logan, 1995).

According to Calame-Griaule (1965), “In the Dogon society, overlap with someone's speech is a serious impoliteness: these words that could not follow their natural way will be repressed in the spleen. The spleen is the seat of grudge and humiliations. Thus, accumulating repressed words can make sick.” Speech has to submit to rules to become an instrument of social communication. In general, “repair mechanisms exist for dealing with errors and violations: stop prematurely or display even rituals.” Overall, turn-taking allows interlocutors to enhance mutual attention and responsiveness (France et al., 2001) which may explain why overlapping/interruption is perceived negatively, preventing the other's turn to occur but also indicating a lack of attention.

Overlapping may also reveal a person's status for example. In his work on Kirundi language in Burundi, Albert (1964) found that the order in which individuals speak in a group is strictly determined by seniority of rank: “the rule for servants, females and other inferiors is to speak when spoken to but otherwise to maintain silence in public.” Leaders talk more than other individuals (France et al., 2001). Men are more likely to interrupt than women which is generally interpreted as a male “power demonstration” but could also be interpreted as reflecting distinct male and female “subcultures” (Maltz and Borker, 1982). Turn-taking shows a level of contextual adaptation: there is for example variability in turn order, turn size, length of pauses according to the number of individuals present (Sacks et al., 1974).

Apart from simple “politeness,” it is obviously difficult to maintain mutual comprehensibility when participants talk at the same time (Duncan, 1972).

In fact, turn-taking is a very general feature of social interactions (games, traffic at intersections …) (Sacks et al., 1974). Turn-taking, “as an orderly distribution of opportunities to participate in social interaction” has been considered, like other such types of interactions, one of the “most fundamental preconditions” for a viable social organization (Schegloff, 2000). According to Sidnell (2001), such rules would correspond to a species-specific adaptation to the contingencies of human interactions, a view shared by Albert (1964) who suggested that this type of interaction is not open to a great deal of cultural diversification. Indeed it is found in a variety of cultures: Thaï (Moerman, 1977), Creols of New Guinea (Sankoff, 1980), Dogon (Calame-Griaule, 1965) amongst others. Although cultural differences are apparent in the duration of pause between turns (the minimal pause under which locutors feel they have been interrupted is about of 0.3 s in France, 0.5 in USA, 1 s in Alaskan Althabascans, Kerbrat-Orecchioni, 2001), avoidance of overlapping appears in all types of languages as well as a minimum gap between turns whatever the languages structure (Stivers et al., 2009). More, within languages, variations in the delay of response are predicted by the same factors such as confirmation or disconfirmation responses or questioner gazing at responder. Indeed, turn taking can be considered as a universal feature within human languages (Stivers et al., 2009).

In all cases, the respect for turn-taking rules requires attention and control and may have evolved over time on the basis of the first rulers who may have been the first to control their vocal production and listen while being listened to MacWhinney (2008). A Dogon saying is that “rules in language = law and order in the society” (Calame-Griaule, 1965).

The universality of turn-taking in humans raises questions about its biological bases and evolutionary trajectory. If it is a species-specific adaptation to social requirements as proposed by Sidnell (2001), there may be either some phylogenetic roots to be found in our closest relatives (non-human primates) or convergence in species with similar social contingencies (Hausberger et al., 2008). Functional convergence (one process of homoplasy) is a widespread phenomenon in evolution, sometimes revealing striking functional similarities between distantly-related species even though the mechanisms involved may be different (Deleporte, 2002). One well known example is birdsong, considered for many years now as the best animal model of language development (Marler, 1970). Amongst the parallels is the observation that both human language and birdsong need to be learned from adult models during development whether in terms of production, perception or usage.

In what follows we will review the turn-taking literature in primates, and the next closest groups which are the non-primate mammals. Then, we will review how birdsong, a flexible type of vocalization, can give rise to vocal interactions whether between group members or territorial or social neighbors.

### Coordination in mammals and birds' vocal interactions

While some aspects of these conversation rules may really be human-specific (e.g., lexical aspects) and difficult to evaluate in animals, other characteristics such as the influence of the number of interlocutors, their social status, “cultural” (interpopulational) differences, the individual's life experience, and the influence of its internal state on the temporal and structural organization of potential turn-taking bouts, can be investigated in animal vocal interactions. Both alternation and overlap (chorus, duets) occur in animal vocal interactions.

In a variety of mammal social species, vocal exchanges occur between a limited number of interlocutors, mostly 2 or 3 (dolphins *Tursiops truncatus*, Janik, 2000; Tyack, 2000; elephants *Loxodonta africana*; Soltis et al., 2005; Campbell's monkeys *Cercopithecus campbelli*, Lemasson et al., 2010).

Non-human primates and other mammals may display the three “classical” forms of temporal organization of vocal interactions (duets, choruses and alternations). Thus, an “organized” *overlapping* is observable when two sperm whales (*Physeter microcephalus*) adjust their timing of “codas” (series of clicks) production (Schulz et al., 2008) or in gibbons who duet by synchronizing their vocalizations (male-female, Geissmann, 2002; mother-daughter, Koda et al., 2013). The extreme case is a chorus where a group joins in calling (e.g., bat spp., Kunz, 1982; Barbary macaques *Macaca sylvanus*, Hammerschmidt et al., 1994; bottlenose dolphins, Kremers et al., 2014; humpback whales *Megaptera novaeangliae*, Au et al., 2000; chimpanzees *Pan troglodytes* Fedurek et al., 2013). *Alternation (antiphony)* is however particularly common in the social call exchanges of different species (bottlenose dolphins, Janik, 2000; elephants, Soltis et al., 2005; Campbell's monkeys, Lemasson et al., 2010; squirrel monkeys, Masataka and Biben, 1987; Diana monkeys, Candiotti et al., 2012; Japanese macaques, Lemasson et al., 2013; bonobos, Touitou et al., in revision; white-winged vampire bats *Diaemus youngi*, Carter et al., 2008; naked mole-rats, Yosida et al., 2007). The structure of sounds is then adapted in that they are often short and produced in sequences with a silent interval, longer than the call itself thus enabling response without overlap. Interval between calls varies according to species (generally 1 s or less but up to 30 s in elephants) and temporal regularities may change within a species: according to call types and their functions (Yamaguchi et al., 2009), to the partner's identity (Biben et al., 1986) and distance (Sugiura, 2007) suggesting an adaptation to the longer latency of response from a more distant partner. The status of the emitter as well as its age are also important for the selectivity of interlocutors within groups. In some species, affiliated individuals exchange more calls (squirrel monkeys *Saimiri sciureus*, Masataka and Biben, 1987; elephants, Soltis et al., 2005; bonobos *Pan paniscus*, Touitou et al., in revision). In other species, the calls of older (Campbell monkeys, Lemasson et al., 2010, Japanese macaques *Macaca fuscata*, Lemasson et al., 2013; marmosets *Callithrix jacchus*, Chen et al., 2009) or higher-ranked (naked mole-rats *Heterocephalus glaber*, Yosida and Okanoya, 2009) individuals will elicit more vocal responses. Individuals can detect and wait for silent windows to vocalize (e.g., cotton top tamarins *Saguinus oedipus*, Versace et al., 2008). This alternation analytic perspective can be extended to non-vocal communication. Gestural signaling sequences can also be considered as interactional projects that develop through courses of action with comparable (<1 s) short delay between requests and responsive moves in both human and non-human primates (Rossano, 2013: Rossano and Liebal, 2014). It has then been proposed that “conversations,” following turn-taking rules, could even be detected in non-human primates (Snowdon and Cleveland, 1984; Symmes and Biben, 1988; Hauser, 1992; Lemasson et al., 2010). Thus, pygmy marmosets *(Cebuella pygmaea)* call in sequence more frequently than expected by chance, while the likelihood of an animal calling twice before the other animal called once was less than expected by chance (Snowdon and Cleveland, 1984). These findings clearly demonstrated that the conversation rules were based on social conventions and that the alternation of calling appeared to be adaptive. This was confirmed recently using a coupled oscillator model revealing dynamics such as those proposed for human conversational turn-taking (Takahashi et al., 2013a). In Japanese monkeys and vervets *(Chlorocebus pygerythrus)*, Hauser (1992) described a decrease of the fundamental frequency before ending a call that could “guide” the turns. He estimated that 1/38 calls were interrupted when the exchange was between adult emitters compared to 6/20 were when the individuals were young. This observation suggests that the ability to respect turns may be acquired during development. This was confirmed by Lemasson et al. (2010, 2011) who showed that young primates are 12 times more likely to interrupt turn-taking by calling twice successively than are adults and by Chow et al. (2015) who demonstrated that common marmoset parents guide vocal turn taking development in their young. In humans, self-monitoring is an essential ability for turn-taking, fully developed only after 2 years of age (MacDonald et al., 2012). In a study on parent-infant vocal interactions in marmosets, it was found that only adults have the capacity to self-monitor their vocal output and avoid call overlap (Takahashi et al., 2013b). According to these authors the neural mechanism underlying the development of self-monitoring could be based on the interactions between three neural structures (representing limbic, motor and auditory regions) with feedback connectivity.

In many species, birdsong occurs mostly in Spring at breeding time and is related to territorial defense and mate attraction (Catchpole and Slater, 1995) and conveys information on individual identity, distance, residency (Falls and Brooks, 1975). It also occurs in the winter flocks and at night roosts for the same species, at a time when they gather in larger groups. In social species, song often occurs all year round and is produced in the context of both intragroup and intergroup encounters (e.g., Brown and Farabaugh, 1997).

In territorial songbirds, networks of neighbors, sharing song structures, can be observed; they also react less aggressively to each other than toward a stranger (Falls, 1982; see Catchpole and Slater, 1995; Briefer et al., 2008) forming a “pseudosocial structure.” Birdsong has long been considered as a male behavior, but in many cases females do sing more than was thought (Riebel, 2003).

Birdsong interactions present a whole range of temporal modalities: alternation is by far the most common form, but duetting and choruses also occur.

**Duetting** is considered as a feature of a pair while chorusing is a group activity (Catchpole and Slater, 1995): in white browed sparrow weavers (*Plocepasser mahali*), the dominant male sings a solo, the dominant pair duets, and the group performs **choruses** (Voigt et al., 2006). Duetting can be antiphonal, or overlapping and synchronized (Hooker and Hooker, 1969; Todt and Hultsch, 1982; Trainer et al., 2002). It seems that most duetting species are monogamous, monomorphic, sedentary and that in about one third of the cases, duetting is antiphonal, one third totally overlapping and one third variable between both (Dahlin and Benedict, 2013). In Australian magpies (*Cracticus tibicen*), choruses occur where the whole social group sings together without clear coordination, in particular in the context of intergroup encounters (Brown and Farabaugh, 1991, 1997). Communal singing is one major characteristic of roosting behavior, where choruses occur before the sleeping phase (Counsilman, 1974). The functions of such communal singing have been suggested to be a synchronization of activities, social bonding, and group or territorial defense (Brown and Farabaugh, 1991; Foote et al., 2008).

**Alternation** is predominant and is based on a singing style that ensures a silent interval after each emission, leaving space for a response (Naguib and Mennill, 2010). In the winter wren (*Troglodytes hiemalis*), 90% of the songs are produced during interactions and the intersong interval is longer when there is a vocal interaction than when the male sings solo (Camacho-Schlenker et al., 2011). Receivers avoid actively overlapping (Wasserman, 1977): in lesser skylarks (*Alauda gulgula*), if two birds start singing simultaneously, one of them stops within 2 s (Gochfeld, 1978, see also nightingales *Luscinia megarhynchos*, Naguib, 1990). In playback experiments, birds often start singing just after the playback in order seemingly to avoid overlapping the next song (Searcy and Beecher, 2011).

**Overlapping** (one bird starts singing before the other has finished, Todt and Naguib, 2000), may occur during these interactions. In general, it stops the exchange: the first emitter falls silent (Schmidt et al., 2006; Naguib and Mennill, 2010). In black capped chickadees (*Poecile atricapillus*), dominant males tend to overlap more which could reflect increased aggressiveness (Ficken et al., 1978; Baker et al., 2012). In robins (*Erithacus rubecula*) and black capped chickadees, overlapping excites the overlapped interlocutor (Dabelsteen et al., 1997; Mennill and Ratcliffe, 2004). It has been suggested that overlapping may be perceived as a directed aggressive signal (Naguib and Kipper, 2005) or even a signal *per se* (Naguib and Mennill, 2010), but more experimental evidence is still needed (Searcy and Beecher, 2009). Alternation in birdsong exchanges suggests turn-taking rules in that the timing allows turns to be taken between two or more interlocutors, and overlapping elicits “irritation” or a rupture of the exchange. However, we do not know how these characteristics are acquired, what their real significance is and how they are influenced by status or bonding. Social structure may be a key factor.

Alternation requires discontinuous songs that leave space for responses and indeed some “true” territorial species with long continuous songs such as skylarks cannot show this alternating pattern (Geberzahn and Aubin, 2014). Alternation appears more in species with distant vocal interactions but social or “pseudo social” types of relationships. Family or very cohesive social groups are more likely to perform choruses.

Many species, such as caciques *Cacicus* sp. (Feekes, 1982; Thieltges et al., 2014), nightingales (Sorjonen, 1983; Naguib et al., 2002), five striped sparrows *Amphispiza quinquestriata* (Groschupf, 1985), great reed *warblers Acrocephalus arundinaceus* (Catchpole, 1983) have two categories of songs that allow both temporal singing styles: a long continuous (often quiet) vocalization often associated with intersexual interactions, and louder, shorter and simpler songs that are more involved in male-male encounters at a distance (Catchpole and Slater, 1995).

In summary, vocal interactions in animals are clearly regulated, especially in terms of timing. Both intra and interspecific variations are observed that hint at possible evolutionary processes: more overlap and communal chorusing in tight social groups, more alternation between distant neighbors, with sometimes both types of exchanges in the same species according to context. There are suggestions that temporal regulation would depend upon both development and social influences.

To date, there is a clear lack of targeted studies on particular animal models where all these facets could be investigated. Very few primate studies and almost no songbird study has considered the context of these different types of exchanges together with developmental issues, and even fewer are devoted to the cognitive (perceptual) processes involved. Comparative work is also often lacking, or draws on species other than those studied in terms of proximate factors. To test the possible social bases for the evolution of temporal aspects such as the turn-taking, we also need to study species from a common phylogenetic lineage, which differ in their social organization.

We will here try and tackle these questions on one songbird species, the European starling *Sturnus vulgaris*, well known for its vocal and social richness, and which has become one of the classical animal models for song studies (e.g., Eens, 1997; Hausberger, 1997). Comparative data from other Sturnid species are now available.

#### Testing turn taking in an animal model: The starling

European starlings are highly gregarious birds that form breeding colonies of a few nests, which can be considered as the basic social unit, especially in sedentary populations (Clergeau, 1989). They forage in flocks from 10 to several hundred birds, and gather in the evening at roosts where several hundred to several thousand birds can be present (Feare, 1984). In all these contexts, song is produced (Adret-Hausberger, 1982). The males spend every morning in their colony (all year round in sedentary populations, in Spring in migratory populations), they visit their nest and have vocal interactions with their colony neighbors, which are generally from 5 to 20 m away. The colony membership tends to be stable over time, although some birds may disappear and be replaced (Adret-Hausberger et al., 1990). Neighbors are therefore familiar. Males defend only the nest vicinity. Vocal interactions between neighbors involve particular vocal structures which are loud simple whistles that are produced with silent intervals between successive whistles, intervals where responses from other birds generally occur (Hausberger, 1991). As in all songbird species, starlings produce both calls and song. Calls are short and simple vocalizations produced in particular contexts for which an immediate function can be identified. Birds produce alarm calls, distress calls or flight calls, for example (Thorpe, 1961). Songs are more complex vocalizations whose functions are not so immediately obvious. Songs are produced in social contexts as well as breeding contexts. Starlings are able to produce two different categories of songs: whistles and warbling (Adret-Hausberger and Jenkins, 1988; Eens et al., 1989). These two categories of song are different in structure and in function as well as in their pattern of acquisition (George et al., 2010). Whistles are short, loud and stereotyped vocalizations that are produced in a discontinuous way. By contrast, warbling is characterized by its complexity and low intensity and consists of successions of motifs (a fixed combination of acoustic elements) produced in unbroken sequences for up to a minute (see also Chaiken et al., 1993).

**Figure 1:**
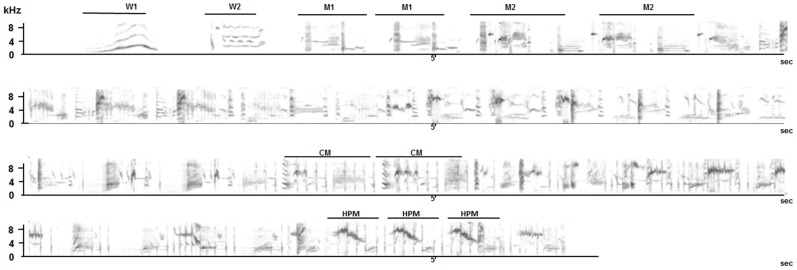
**Song sequence of a wild starling**. The typical song sequence starts with two different types of whistles (W1 and W2). The warbling sequence starts with variable motif types (M1, M2 etc…) that can be repeated several times. Click motifs (CM) appear in the middle of the sequence. High pitched trill motifs (HPT) are characteristic of the end of the sequence.

Whereas whistles can be produced independently, warbling is often preceded by whistles and it then shows a clear organization based on repetition of motif types and an increase in tempo and frequency ending with clicks and followed by high-pitched trills (Figure 1). Warbling is not used in alternating vocal interactions and is mostly sung solo in the field. Playback experiments show that the birds react to whistled structures by replying vocally while they do not respond nor change their behavior when warbling (pers. obs). The developmental course of these two categories of songs is different (Poirier et al., 2004; Bertin et al., 2007). Warbling develops progressively from subsong in the course of the bird's first year of life, whereas whistles appear suddenly during the first winter around 9 month of age (Adret-Hausberger, 1989). Moreover, young birds raised without direct contact with adults will not develop whistles but will produce warbling song (Poirier et al., 2004; Bertin et al., 2007). Finally, neuroethological as well as functional magnetic resonance imaging (fMRI) studies performed on starlings revealed that these two distinct categories of song are not processed in the same way in the brain (George et al., 2008; De Groof et al., 2013).

Here we will focus on the singing style that emerges from the use of one or the other of these song categories, one discontinuous and enabling alternating interactions, the other continuous and hence not appropriate for turn-taking types of interactions.

One other interesting feature is that these two categories of songs have been found in other Sturnids such as the Indian hill mynah *Gracula religiosa* (Bertram, 1970) or the wattled starling *Creatophora cinerea* (Sontag, 1991) suggesting that comparative studies within this family of songbirds could be promising for understanding the evolutionary roots of the temporal regulation of vocal interactions.

In the following section, we describe a series of observations and experiments on the European starling, followed by field data on other sturnids, in order to examine the different facets of temporal regulation of interactions in one species in relation to the four questions of Tinbergen (1963): causation (why do these temporal features appear now and how are they processed?), ontogeny (how did they develop at the individual level?), function (what are their immediate functions?) and evolution (what adaptations led this species to develop these forms of interaction?).

### Promoting or not turn-taking: Does the social situation influence temporal features of song?

Here we compared the singing style of European starlings living in colonies at different social densities.

#### Methods

Song recordings from 21 birds were re-analyzed in order to examine their singing styles (warbling/whistles). Data were available for four adult males recorded in isolation in captive conditions and 17 adult males recorded in the morning near their nests in the field. Recording sites and dates are shown in Table 1. More details about the recording conditions are given in the references mentioned. Additional aviary recordings when in a large mixed group were also available for the 4 isolated birds (Hausberger et al., 1995) (Table 1).

**Table 1:** **Recording sites, social conditions for all the birds**.

**Birds**	**Captive birds**	**Wild birds**									
	**CM1-CM4**	**WM1**	**WM2**	**WM3**	**WM4**	**WM5**	**WM6**	**WM7-WM8**	**WM9-WM11 WM12-WM13 WM14-WM17**		
Social conditions	Isolation	1 nest	1 nest	1 nest	1 nest	2 nests	3 nests	3 nests	6 nests Colony 1	11 nests Colony 2	18 nests Colony 3
Sites	Rennes (F)	Auckland (NZ)	Seewiesen (Germ)	Slimbridge (G.B)	Rennes (F)	Rennes (F)	Rennes (F)	Nouvoitou (F)	Rennes University Campus (F)		
Pairing Status	None	Unpaired	Paired	Paired (2 0)	Paired	Paired	Paired	Paired	Paired		
Dates recordings	January 1992	Spring 1987	Spring 1988	March 1990	March 1989	March 1992	March 1990	March 1979	March 1979 March 1982		
References	Hausberger et al., 1995	Adret-Hausberger and Jenkins, 1988	Unpubl.	Hausberger and Black, 1990	Unpubl.	Unpubl.	Henry et al., 1994	Hausberger and Guyomarc'h, 1981	Hausberger and Guyomarc'h, 1981; Adret-Hausberger, 1986		

All field recordings were made in the morning during the first hours of daylight or during the two last hours when song is most frequent during spring in the breeding colony. Most birds were paired (most recordings are from sedentary populations). We recorded only adult males which had visited a nest, and were singing close to their nest. These recording sessions lasted one to several hours. The colony size was noted: a bird nesting singly or in colonies of 2, 3 up to 18 nests. Two nests were considered as belonging from different colonies when they were more than 200 m. apart (Hausberger and Guyomarc'h, 1981). Since colonies of 6–8 nests, 9–11 nests, 12 and 13 nest and 14–18 nests showed the same trends and the number of such colonies was low, we pooled the corresponding data. The captive males had been caught on Jersey Island. They were kept in sound proof chambers at day lengths corresponding to the natural photoperiod. Birds had water and food *ad libitum* (commercial pellets for turkeys, and apples). Recordings were made continuously for 4 consecutive days for each isolated bird. Recordings were made using different tape, or cassette- recorders and microphones (see references). Sound analyses were carried out on an Amiga microcomputer (Richard, 1991). We considered that different elements belonged to the same song bout when they were separated by less than 20 s. This was based on data on whistled sequences showing that successive whistles within a sequence can be separated by up to 12 s (Hausberger, 1991). A warbling sequence corresponds to a succession of elements separated by less than 1 s (Adret-Hausberger and Jenkins, 1988; Hausberger, 1997). Since different studies are summarized here, the recording times were different for the different birds and therefore the absolute number of bouts, warbling or whistle sequences could not be compared between birds. This study was carried out in accordance with the recommendations of European Communities guidelines (European Communities Council Directive of 24 November 1986 (86/609/EEC). The protocol was approved by the local Ethic Committee in Animal experiment of Rennes (CREA-07).

#### Results

##### Individual adaptations to the social situation (Appendix 1 in Supplementary Material)

For four males, their song had been recorded both when in a mixed group in an outdoor aviary and while they were in isolation in sound proof chambers When isolated, these males produced songs that included at least one warbling sequence whereas whistles were not always present. Almost all (*X* = 92.53 ± 7.89%) whistle sequences were followed by warbling, which was also generally preceded by a whistle (see Appendix 1 in Supplementary Material). The whistle sequences were composed of a very low number of whistles (mostly 1–3) (compare to Hausberger, 1991). The proportion of warbling and whistle sequences was similar for two birds but the two other birds showed a higher number of warbling sequences than whistle sequences. Thus, overall, warbling clearly predominated in this context, given also its longer time duration.

When the same birds were observed in a group, they showed a lower proportion of sequences including warbling (*Xi* = 98.1 ± 9.95, *Xg* = 65.42 ± 11.35, Fisher test for the 4 males, *p* ≤ 0.05), a lower proportion of whistle sequences followed by warbling (*Xi* = 92.53 ± 7.89%, *Xg* = 55.95 ± 15.31, Fisher test, *p* ≤ 0.05 for three out of the fourmales) and also a lower proportion of whistles per sequence (*Xi* = 1.98 ± 0.48, *Xg* = 1.51 ± 0.29, *t*-test, M1 *p* ≤ 0.05 for the four individuals) (see Appendix 1 in Supplementary Material).

##### Isolation vs. field recordings (Table 2 and Appendix 1 in Supplementary Material)

Compared to the songs of isolated birds, field recordings revealed a lower proportion of sequences including warbling (Mann Whitney, *n*_1_ = 4, *n*_2_ = 17, *U* = 0, *p* < 0.002), a lower proportion of whistled sequences followed by warbling (*U* = 0, *p* < 0.002) and a lower proportion of whistles followed by warbling (*U* = 4, *p* < 0.05) while the number of whistles per sequence was lower in isolation (*U* = 1 *p* < 0.002). The proportion of whistle sequences compared to warbling sequences was overall much higher in the field as well as the ratio of the whistle sequences and warbling sequences (*U* = 2, *p* < 0. 001) in both cases. Isolated birds and wild birds in all situations did produce the same proportion of song bouts including at least one whistle (*U* = 32, *p* > 0.05).

**Table 2:** **Song characteristics of each individual**.

**Birds**	**WM1**	**WM2**	**WM3**	**WM4**	**WM5**	**WM6**	**WM7**	**WM8**	**WM9**	**WM10**	**WM11**	**WM12**	**WM13**	**WM14**	**WM15**	**WM16**	**WM17**
Total number of song bouts	120	47	97	62	49	52	74	23	32	45	26	47	31	24	27	25	35
Total number of whistles	331	130	146	103	112	126	198	62	124	268	58	346	162	135	158	208	211
Proportion bouts including warbling % (Number)	67 (81)	77 (36)	60 (58)	84 (52)	55 (27)	40 (21)	61 (45)	57 (13)	53 (17)	40 (18)	58 (15)	53 (25)	17 (5)	8 (2)	7 (2)	12 (3)	29 (10)
Proportion bouts including whistles % (Number)	92 (110)	100 (47)	80 (78)	69 (43)	82 (40)	87 (45)	82 (61)	91 (21)	81 (26)	96 (43)	77 (20)	98 (46)	97 (30)	92 (22)	100 (27)	100 (25)	89 (31)
Proportion whistle sequences followed by warbling % (Number)	65 (71)	77 (36)	50 (39)	80 (33)	42 (18)	31 (14)	52 (32)	52 (11)	42 (11)	37 (16)	45 (9)	52 (24)	13 (4)	0 (0)	7 (2)	12 (3)	19 (6)
Number whistle per sequence X ± sd	3.1 ± 1.9	2.7 ± 1.8	2 ± 1.5	2.5 ± 1.8	2.4 ± 1.6	2.9 ± 1.7	3.2 ± 3.3	2.9 ± 2.9	4.8 ± 4.4	6.2 ± 4.6	2.9 ± 1.8	7.5 ± 5.4	5.4 ± 4.2	6.1 ± 6.0)	5.9 ± 3.7	8.3 ± 8.4	6.8 ± 6.2
Proportion of whistles followed by warbling %	21	28	27	32	16	11	16	18	9	6	18	7	2	0	1	1	3
Proportion warbling preceded by whistle	88	100	67	63	67	67	71	85	65	89	60	96	20	0	100	100	60
Number whistle sequence/warbling sequence	1.3	1.3	1.3	0.8	1.6	2.1	1.4	1.6	1.5	2.4	1.5	1.8	6	11	13	8.3	3.1

##### The importance of colony size (Table 2 and Figure 2)

Clear differences appeared in the singing style of birds according to colony size. As colony size increased, we found:

- a decrease in the proportion of bouts including warbling (Spearman test, *N* = 17, *r*_*s*_ = −0.89, *p* < 0.0004) in relation to colony size (Kruskall Wallis test, *H* = 12.5, *n*_1_ = 4, *n*_2_ = 4, *n*_3_ = 3, *n*_4_ = 6, *p* < 0.0006).
- a decrease in the proportion of whistle sequences followed by warbling (*r*_*s*_ = −0.81, *p* = 0.001) with differences according to colony size (*H* = 9.8, *p* < 0.02).
- a decrease in the proportion of whistles followed by warbling (*r*_*s*_ = −0.89, *p* = 0.0002) with differences according to colony size (*H* = 12.9, *p* < 0.005).
- an increase in the mean number of whistles per sequence (*r*_*s*_ = 0.83, *p* = 0.001) with differences according to colony size (*H* = 10.5, *p* = 0.02).
- an increase in the ratio of the number of whistle sequences to the number of warbling sequences (*r*_*s*_ = 0.9, *p* = 0.003) with differences according to colony size (*H* = 13.1, *p* = 0.004) (Figure 3).

**Figure 2:**
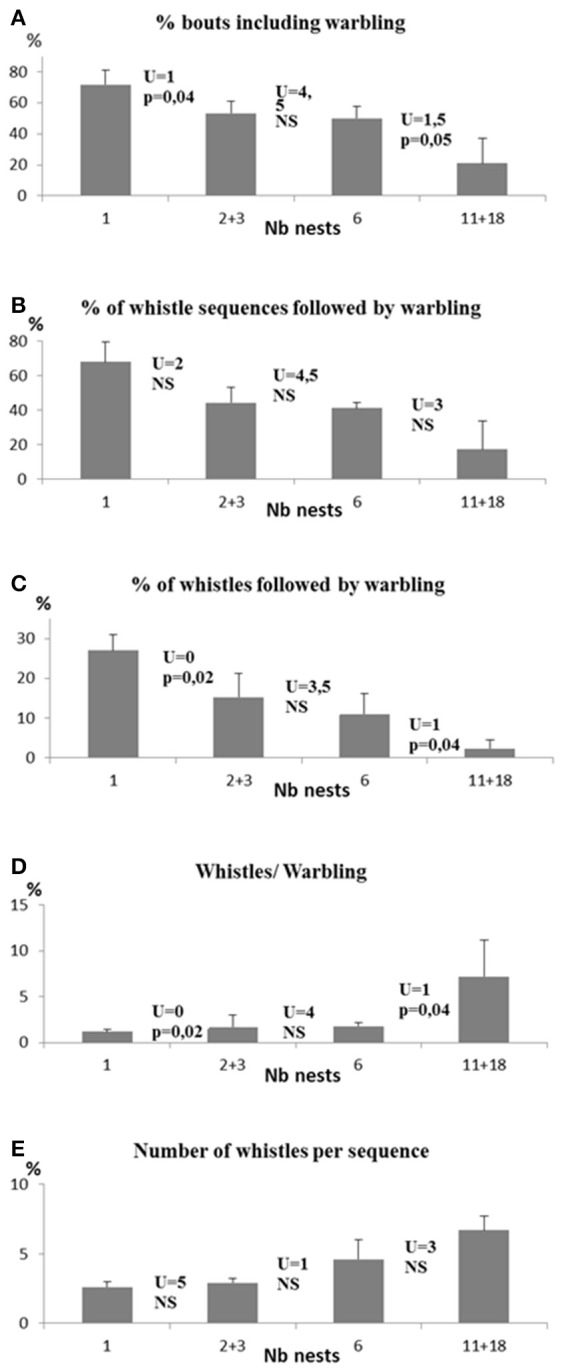
**Differences in song characteristics according to the size of the colony. (A)** % of bouts including warbling; **(B)** % of whistle sequences followed by warbling; **(C)** % of whistles followed by warbling; **(D)** ratio Whistles/Warbling; **(E)** number of whistles per sequence.

**Figure 3:**
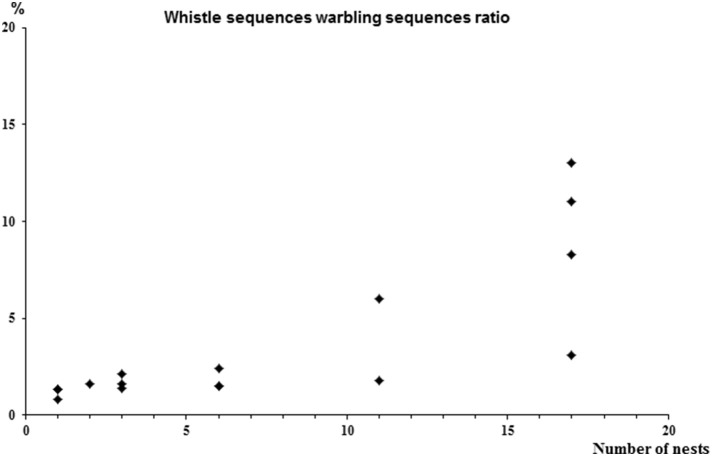
**Correlation between the number of whistle sequences and the number of warbling sequences (***r***_***s***_ = 0.9, ***p*** = 0.003)**.

While all these comparisons were verified when colony sizes were compared pairwise, the colony sizes 2/3 and 6 nests did not show significant differences in most of the above mentioned comparisons. Additional differences were observed between colony size 1 and 11/18 for the percentage of whistles following by warbling and the number of whistles per sequence (Mann Whitney *U* = 1, *P* < 0.003 and *U* = 0, *p* < 0.005 respectively) (see Figure 2) suggesting that the overall trend is accentuated in extreme social situations.

#### Conclusion

**Figure 4:**
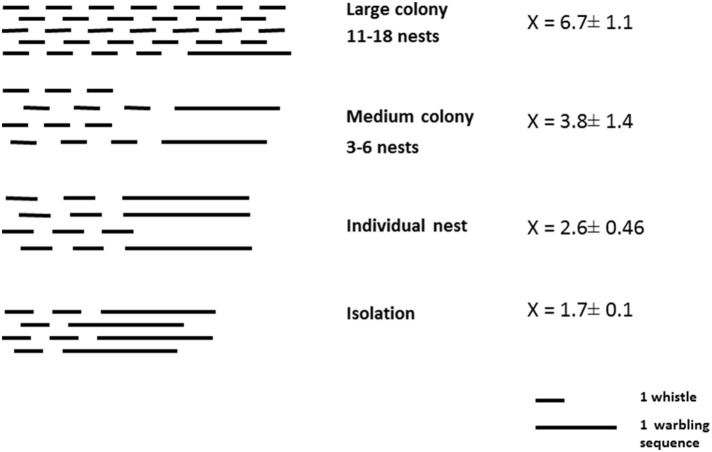
**Song style of birds belonging to colonies of different size**. Although the birds were recorded in very different conditions, a clear trend appeared toward an increase in whistling (hence discontinuous songs) and a decrease of warbling (hence continuous song) with increasing colony size (= number of neighbors) (From Hausberger, 1997).

Singing style is clearly influenced by the social situation in male starlings. The more birds there are around them, especially in the breeding context, the more they favor the production of discontinuous songs, which is a prerequisite for alternating vocal exchanges. In large colonies, male starling song showed a high proportion of whistles, leaving much opportunity for interactions and transfer of information between neighboring males (Figure 4). Data from breeding sites where the birds nested singly were similar to those obtained in isolated captive birds, revealing that it is more the presence of potential vocal partners than the presence of another bird (mate) that influences the choice of a singing style. Comparison of the same birds in different contexts revealed that there is an individual capacity to adjust the singing style to the social situation.

### Are there temporal regularities in starling vocal interactions?

Here the immediate responses of male starlings in terms of temporal opportunities for response in the presence of another individual and its interactional status were observed in spontaneous interactions.

#### Methods

Seven male starlings were observed in the same breeding colony (4 in 2002, 3 in 2003) between March 17th and 27th 2002 and between March 17th and April 15th in 2003 from 7 am (sunrise) to 11 a.m. All were paired at that time of the year. The colony was composed of 5–6 pairs. This study was carried out in accordance with the recommendations of European Communities guidelines (European Communities Council Directive of 24 November 1986 (86/609/EEC). The protocol was approved by the local Ethic Committee in Animal experiment of Rennes (CREA-07).

Each full whistle sequence of the focal bird was recorded until it started warbling or left. Two contexts of singing were considered: 1- singing alone with no congener present in the vicinity and 2- singing with another male silent or singing in the vicinity.

Song recordings were made using a Sony TC D5 cassette recorder and a Sennheiser directional microphone (MZA 14 P48) in 2002, or a Sony microphone (EMC 144) fixed on a polyester parabola in 2003. Vocalizations were analyzed using a computer (Unix Silicon Graphics Ind), and a custom-designed sound analysis software (ANA, Richard, 1991).

#### Results

**Figure 5:**
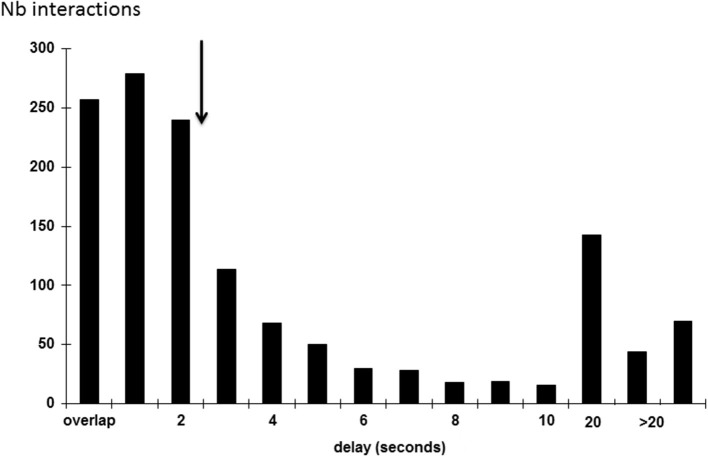
**Interval separating two successive whistles produced by two different individuals (overlap: when two whistles overlap)**. The arrow indicated a break in the interactions after a 2 s delay.

We plotted the intervals between successive whistles produced by two different birds (Figure 5). More than half of the whistles (56.4%) were produced within 2 s. We thus considered that two whistles separated by 2 s or less belonged to a single vocal interaction (see also Adret-Hausberger, 1982; Miller et al., 2004). Eight hundred and thirty five whistles were recorded in total.

**Figure 6:**
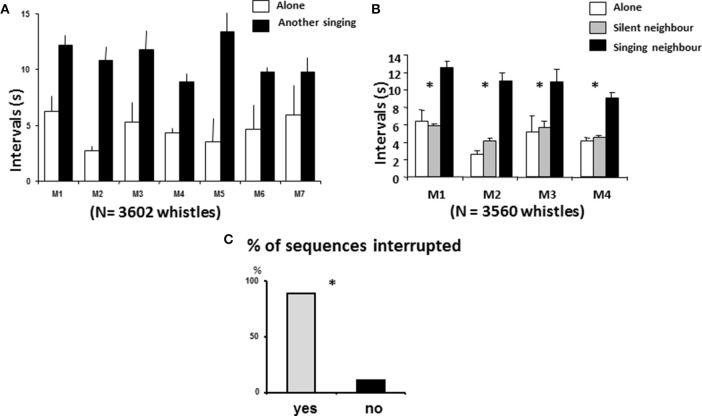
**Song behavior according to immediate context. (A)** Males increased their interval duration when another bird was singing (Wilcoxon, *N* = 7, *T* = 0, *p* < 0.02). **(B)** Birds did not change their interval duration when another bird was present but silent and increased their interval duration when the other bird was singing. **(C)** Most of the birds interrupted the vocal interaction in case of overlap (^*^:*X*^2^ = 2256, *df* = 1, *p* ≤ 0.05).

The intra-individual interwhistle interval (IWI) clearly increased when another starling was singing nearby (*Xa* = 4.7 ± 1.3 s, Xns = 11 ± 1.6 s Wilcoxon, *N* = 7, *T* = 0, *p* < 0.02 (Figure 6A). Indeed, five of the seven males doubled this interval and one quadrupled it.

For four of the birds, we recorded sessions when the neighbor was silent: clear differences appeared again: the IWI did not differ significantly between the solitary situation and the “silent neighbour” situation (*Xa* = 4.3 ± 1 s, Xnst = 5.11 ± 0.65 s, Mann Whitney, *n*_1_ = *n*_2_ = 4, *U* = 5, *p* > 0.5 while the IWI in the “singing neighbour situation” differed from both (Xns = 10.68 ± 0.93 s, MW; alone/neighbor singing, *U* = 0, *p* ≤ 0.05 in both cases) (Figure 6B). Only 133 instances of overlapping (second emitter started before the end of the whistle) were observed, but in 83% of the cases they were associated with the end of vocal exchanges (first emitter became silent or flew away), which is more than expected by chance (*X*^2^ = 63.11, *df* = 1, *P* ≤ 0.001) (Figure 6C).

#### Conclusion

It appeared that starlings take into account the social context when they are singing. By increasing interval duration between two whistles, starlings clearly leave space for other birds to reply and therefore make turn-taking possible. Another element that showed evidence of “conversation rules” in the starling was a response overlap between whistles from two males, which appears here as “breaking the rule” and led to the end of the exchange.

### Developmental issues: How do young birds acquire an appropriate singing style?

The impact of developmental conditions, both on the sensory and social levels, has been tested through a series of experiments.

#### Normal development

Young starlings like other songbirds develop their songs slowly with distinct stages, starting with “subsong,” at the age of about 3 months: a long, continuous, disorganized vocalization where the young bird is just practicing, and then a plastic stage where elements of the future song appear progressively. It has been suggested that subsong and plastic song are analogous to infant babbling (e.g., Marler, 1970).

Also like other songbirds, starlings need to hear adult song in order to develop normal songs (e.g., Chaiken et al., 1993). Little attention has been paid in the developmental studies of starling song or even other songbirds to how developmental stages might affect turn-taking responses.

**Figure 7:**
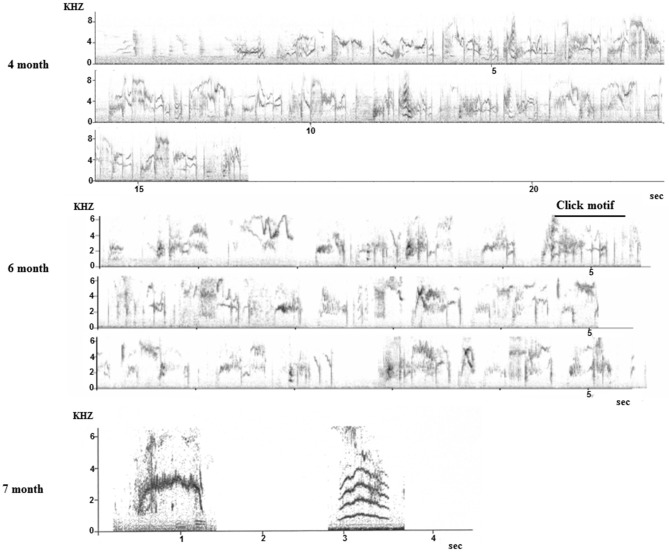
**Sonograms of song produced by young starlings during the first year**. **Top**: subsong produced during the first summer (4 months old). **Middle**, sequence produced at 6 months: click motifs are recognizable. **Bottom**: two whistles recorded at 7 months old.

Field observations are almost impossible as the young birds disperse and become nomadic after fledging (Feare, 1984), thus only some data from captive birds are available (they are also difficult to breed in captivity). Monitoring nine young males from birth to adulthood in an aviary where they were kept with their parents confirmed anecdotal reports from the field in terms of the timing of subsong and plastic song but also revealed that the first whistles (hence discontinuous songs) were produced in November, at the age of 7 months. Until then, only continuous song was produced although the plastic song starts showing some disruption (Figure 7).

Another pilot study on one young male raised without adults but able to hear adult birds showed the same trend with the first whistles appearing at the age of 9 months (Adret-Hausberger, 1989). In all cases, the whistles appeared suddenly and quite independently from subsong that seemed to develop progressively into the adult warbling. We noted that the first click motifs appeared in the subsong at 6 months and subsong sequences progressively showed more adult-like organization (Figure 7). It has been proposed in starlings as in other songbirds that warbling types of songs (long and continuous) could be an adult form of subsong (Adret-Hausberger, 1989).

#### Disturbed ontogeny: The importance of adults

##### Sensory and physical deprivation of experience with adults

Existing data on starlings raised without exposure to adult song or contact with adult birds were reanalyzed in order to extract information on their singing style. Four male starlings were taken as nestlings (2–5 days old) and hand raised without any contact with adults. They were kept respectively in groups of inexperienced animals: 1 male with 4 females of the same age (May 1993), 2 males and one female of the same age (May 1992) and one male amongst other clutches of 19 other males and females (May 1981). Their song was recorded when adult at 1 year old. This study was carried out in accordance with the recommendations of European Communities guidelines (European Communities Council Directive of 24 November 1986 (86/609/EEC). The protocol was approved by the local Ethic Committee in Animal experiment of Rennes (CREA-07).

**Figure 8:**
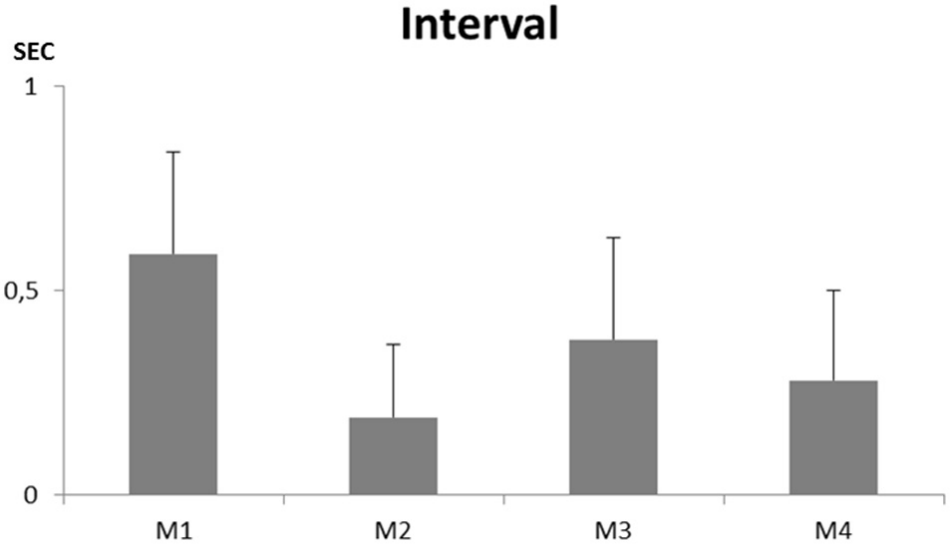
**Intermotif intervals recorded for four young male starlings raised without exposure to adult song or contact with adult birds**.

Forty-five to 123 song sequences could be recorded from each individual. None of them ever produced a whistle. They all sang a continuous song that showed some similarities to a “normal adult warbling” especially in its continuous type of structuring (Figure 7). While separate motifs appear, the intermotif intervals were, as in a normal adult song (e.g., Eens et al., 1989) too short to permit a non-overlapping response from another bird (*X* = 0.19 ± 0.18 to 0.59 ± 0.25 s) (Figure 8).

##### Varying the type of contact with adults (Poirier et al., 2004)

**Figure 9:**
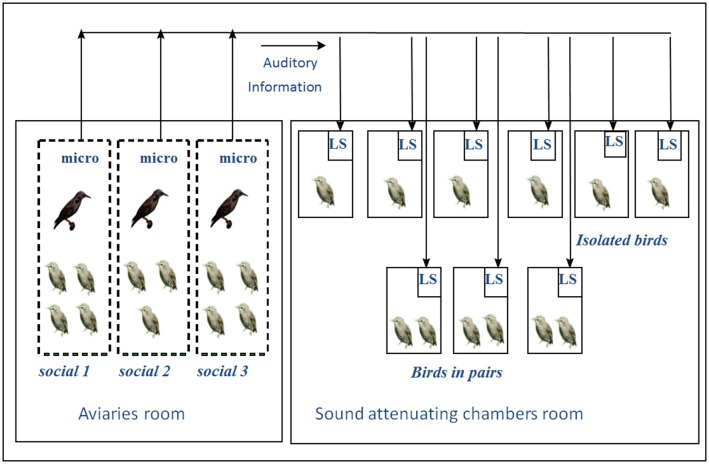
**Experimental set-up: In the aviary room, young birds housed in the three different aviaries could hear and see each other**. In the soundproof chambers, birds were housed individually or in pairs. All the experimental birds received the same auditory exposure. Birds in the soundproof chambers could hear all the songs from the birds in the aviaries via microphones and loudspeakers. (M, wild males; m, experimental males; f, experimental females; LS, loudspeaker; micro, microphone; →, direction of auditory information transfer (from Poirier et al., 2004).

This experiment involved 26 young starlings taken from the nest in April 1998 when 2–5 days old and then hand raised for 2 months. In June 1998, they were placed in one of three situations: eleven (5 males) were placed in groups of 3 or 4 in three aviaries together with wild caught adult males in indoor aviaries; 6 (4 males) were kept in isolation and 6 (4 males) in pairs of inexperienced birds in sound proof chambers fitted with loudspeakers that transmitted the sounds from the aviary room (Figure 9). The isolated and pair raised animals could thus continuously hear the vocal interactions that occurred in the aviaries. This study was carried out in accordance with the recommendations of European Communities guidelines (European Communities Council Directive of 24 November 1986 (86/609/EEC). The protocol was approved by the local Ethic Committee in Animal experiment of Rennes (CREA-07).

Their song was recorded when they were adults in 1999 after 1 year spent in this situation. The recordings show that all 5 males raised in direct contact with an adult produced whistles while of the others, only the two isolated males did so. They were also those whose output showed the closest resemblance to adult song. The pair-raised animals did not produce any discontinuous song, hence separate whistles. They did produce some whistled notes but these were included in a warbling sequence with no time interval. They also had a very variable warbling song much like juvenile subsong.

Because they had no other sensory stimulation, isolated birds paid more attention to the adult song heard through the loudspeaker and hence developed some discontinuous songs (Poirier et al., 2004). It remains to be established if they will use them in an appropriate way. Further studies seem to indicate that the absence of adult contact during development prevents the development of a normal singing style and proper use of song types (George et al., 2010).

Appropriate social contact during development is thus necessary and crucial in order to produce songs that enable an alternating communication pattern. The birds raised in the aviaries with one adult model nevertheless still showed some abnormalities that pose questions concerning the importance of the adult-young ratio. They formed mostly small same-sex age groups that sang together with mainly overlapping vocal interactions.

##### Testing the impact of the adult-young ratio (Bertin et al., 2007)

Twenty male starling nestlings (6–8 days old) were taken from the nest in April 2002. They were hand raised and were kept as a mixed social group with 27 peer females until the age of 2 months with no contact with any adult. In June, they were allocated to three different rearing conditions: (1) dyadic: one adult-one young, (2) group tutored: 7 young and 2 adults, (3) group: 5 young birds together. The groups could hear but not see the other animals (which were housed in the same room), thus providing a similar auditory environment. This study was carried out in accordance with the recommendations of European Communities guidelines (European Communities Council Directive of 24 November 1986 (86/609/EEC). The protocol was approved by the local Ethic Committee in Animal experiment of Rennes (CREA-07).

The song of the experimental animals was recorded and analyzed when they were 1 year old. The results show that the repertoire of whistles followed a gradient with fewer whistle types in the group tutored than in the dyadic situation and almost no whistles produced by the peer-only group (only 1 whistle type in two of them).

When still in their developmental setting, both groups (group tutored or not tutored) sang more (in time) than the animals placed in the dyadic situation, but since their song repertoire was mostly or only composed of warbling, they kept singing together, overlapping without any temporal organization.

### Brain mechanisms and plasticity: The processing of song categories and the effect of experience

It was hypothesized that if the two different song categories (discontinuous/continuous) had a different functional significance and as shown above, different developmental trajectories, the brain processes involved should be to some extent different. The following studies were carried out in accordance with the recommendations of European Communities guidelines (European Communities Council Directive of 24 November 1986 (86/609/EEC). All the protocols were approved by the local Ethic Committee in Animal experiment of Rennes (CREA-07).

#### Song processing in wild caught adults (George et al., 2004, 2008)

In a series of experiments on the processing of starling song in the brain, we tested the electrophysiological responses of field L (primary auditory area) and NCM (secondary auditory area) neurons of awake restrained adult (wild caught) starlings while they were exposed to a variety of species specific sounds (whistles, warbling elements) and artificial sounds (white noise, pure tones). Using a systematic approach to record neuronal activity (George et al., 2003), we were able to record the activity of almost 3000 neurons in the Field L and 2000 neurons in the NCM from 6 individuals each time.

It appeared that the distribution and level of response respectively varied according to the song category. There was lateralization of song processing so that in Field L, the whistles were processed more in the right hemisphere while the warbling was processed mostly in the left hemisphere, revealing a differential processing of these two categories of songs. In the NCM, which as a secondary area, processes more complex associative information (e.g., Chew et al., 1996), it appeared that most neurons responded first of all to songs bearing individual information, but both the proportion of responsive neuronal sites and the magnitude of the neuronal responses differed according to the functional song classes. A gradient of response was observed from the class 1 whistle (eliciting the lowest level of responses), to the class 2 whistles and then warbling which clearly triggered more responses than the two classes of whistles.

#### Conclusion

Since the brain processes functional categories of songs differently and at different levels, it may trigger appropriate vocal production and enable the bird, when hearing one song category, to rapidly “decide whether or not” to reply.

#### How can social experience during development affect brain processing of song categories? (Cousillas et al., 2004, 2006; George et al., 2010)

Responses of field L neurons of adult starlings raised without adults (no sensory contact) using the same procedure as above has revealed that the whole area (Field L) lacks the typical spatial organization of normal adults and also the typical neuronal selectivity toward specific song elements (Cousillas et al., 2004).

Social experience *per se* can evidently have as much influence on the development of the primary auditory area as the sensory experience in the experiment by Poirier et al. (2004). Thus, both the birds raised in pairs or solitarily showed as many abnormalities (lack of neuronal selectivity) as the sensory deprived birds. The lack of contact with adults was obviously sufficient to prevent proper development. Another intriguing finding was that even the birds raised in a group with one adult showed deficiencies, which seems to reflect their lack of social bonding with the adult (Cousillas et al., 2008).

**Figure 10:**
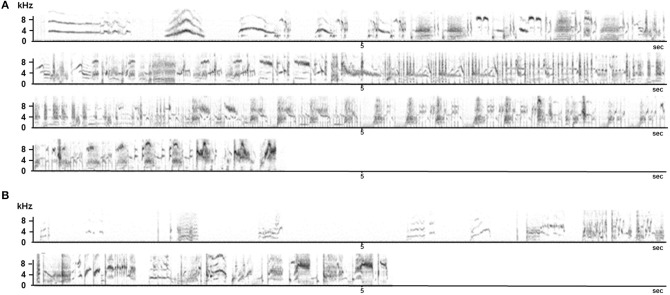
**Song sequences produced by an adult (A) and by a 2 year old birds that did not receive adult tutoring (B)**. Recordings were made at the same time of year.

Similar findings were obtained at the NCM level: 10 young birds were taken from the nest, hand raised, and then placed in a large outdoor aviary where they could hear wild adults but had no direct contact with any adult. Four months later they were transferred as a group to an indoor aviary with no auditory nor direct contact with adults for 12 months. These birds, when adult, had a fairly normal song repertoire including whistled and warbling structures. However, they did not produce sequences of whistles as “normal” starlings do (Hausberger, 1991), and placed them within warbling sequences which made them inappropriate for alternating vocal interactions (Figure 10). Interestingly, the electrophysiological recordings of the NCM neurons showed a clear deficiency in processing song categories (George et al., 2010). The lack of direct experience with adults despite a rich auditory experience therefore induced a singing style that did not promote alternation in vocal interactions despite the production of appropriate structures. Since brain processes devoted to song categorization were clearly affected, the birds probably could not recognize appropriate times for replying.

#### Conclusion

Social bonding and hence selective attention may be a key factor in developing the necessary brain processes and therefore the ability to communicate in an appropriate way.

### Turn-taking as a social adaptation: An evolutionary process?

In the Eastern Cape in South Africa, four species of starlings with different social systems offered an opportunity to test the hypothesis that the temporal regulation of vocal interactions would reflect their social organization. The red-winged starling *Onychognathus morio*, pale-winged starling *Onychognathus nabouroup*, African pied starling *Spreo bicolor* and Cape glossy starling *Lamprotornis nitens* are widely sympatric in the region, but range from solitary pairs through colonial groups to communally-breeding species (Feare and Craig, 1999; Craig and Feare, 2009):

- A territorial species: *Onychognathus morio*

The red-winged starling is a sedentary species. Monogamous pairs remain together for at least three successive seasons and are associated throughout the year (Rowan, 1955; Craig et al., 1991). During the breeding season (October—March), pairs are restricted to their breeding territory (approximately 200 m^2^) and very rarely join the flocks of non-breeding birds. Breeding pairs are extremely territorial and intraspecific aggression is very common. During the non-breeding season, both pairs and non-reproductive birds gather in flocks of varying sizes and spend the night together in large roosts (Craig and Feare, 2009).

- A colonial species: *Onychognathus nabouroup*

Monogamous pair bonds of the pale-winged starling are maintained throughout the year, and the birds apparently remain together for several seasons. At the beginning of the breeding season, male and female defend a small area around the nest, but pale-winged starlings seem clearly less aggressive than red-winged starlings. Throughout the year, birds roost in small flocks in groups on cliffs, with breeding pairs generally roosting at their nest site (Craig et al., 1991).

- A “familial” species: *Lamprotornis nitens*

Cape glossy starlings, a mainly sedentary species, breed in small family groups. Several monogamous couples nest in a same site (September—February). Nests may be in tree holes or other structures, and the same site is often re-used in successive years. According to Craig (1983) and Craig and Feare (2009), up to three birds, mostly young non-reproductive birds, help pairs to care for nestlings. During the non-breeding season, birds may gather in larger flocks of 10-20 birds to forage and share a regular roost site.

- A “communal” species: *Spreo bicolor*

Throughout the year, African pied starlings live in flocks of 15–25 individuals. Stable monogamous pairs re-use the same nest sites in successive breeding seasons (September—January). Several nests can be found close to each other in burrows or holes. During the breeding season, up to seven helpers can feed the young with the parents, and helpers may feed young at three different nests during a single breeding season (Craig, 1987). During the non-breeding season, pied starling groups may be nomadic and join other groups at communal roost sites (Craig and Feare, 2009).

#### Methods

This study was conducted in the Eastern Cape region in South Africa where the four species occur, often at the same sites. Songs have been recorded since 2003, mainly during the breeding season. This study was carried out in accordance with the recommendation of European Communities guidelines (European Communities Council Directive of 24 November 1986 (86/609/EEC). The protocol was approved by the local Ethic Committee in Animal experiment of Rennes (CREA-07).

Red-winged starling vocalizations were recorded primarily on Rhodes University campus in Grahamstown and in the vicinity (2003–2004). Pale-winged starling vocalizations were recorded at one site: Graaff-Reinet (2005). Pied starling vocalizations were recorded at three sites: Table Farm, Queenstown and Graaff-Reinet (2003 to 2005 and 2008). Finally, glossy starling songs were recorded at five sites: Thomas Baines Nature Reserve, Table Farm, Salem, Kariega Private Game Reserve (all in the Grahamstown area) and Queenstown (2003–2004).

From 2003 to 2005, a Sony TC-D5 Pro II tape recorder and a micro-directional microphone Sennheiser MKH 70 P48 were used to record vocalizations in the field. After 2005, we used a digital recorder Marantz PMD 660 and a directional microphone Sennheiser MKH 416 P48 (recordings made in 44.1 kHz/16 bits).

Most recordings were obtained in the morning (6–10 a.m.), and in the hottest hours of the day (12 a.m.–15 p.m.), corresponding to the peaks of activity of the studied birds (Feare and Craig, 1999). According to Fry et al. (2000), both sexes sing in all four species, despite the fact that, except for *O. morio*, males and females are not distinguishable. Vocalizations were analyzed using homemade software for song analyses (ANA, Richard, 1991). The amount of song recorded is summarized in Table 3.

**Table 3:** **Song recordings for the four South African starling species and their song characteristics: temporal features (durations in seconds, mean ± SD); proportions of discontinuous songs (DS) and continuous songs (CS)**.

	***O. morio***	*****O. nabouroup*****		*****L. nitens*****		***S. bicolor***
Number of individuals	45	9		30		16
Total time of analyzed song (min)	6000	41		184		70
Total number of motifs analyzed	4500	1021		11±000		206
DS and CS song proportion (%)	**DS**	**DS**	**DC**	**DS**	**CS**	**CS**
	100	16.54	83.46	5.43	94.57	100
Motif duration	0.76±0.23	0.15±0.001	0.3±0	0.32±0.06	0.29±0.04	0.17±0
Phrase duration	0.76±0.23	0.15±0.001	1.79±0.56	0.7±0	2.91±4.29	3.19±2.07
Number of motifs per sequence	1±0	1±0	5.67±1.91	2±0	6.92±3.12	11.78±7.89
Duration between motifs	>1	>1	0.08±0.04	0±0	0.2±0.04	0.13±0.03
Duration between sequences	8.96±4.58	2.99±1.49	7.19±3.68	4.07±4	2.28±1.20	2.06±0.8

Here we focused our analyses on the temporal aspects of songs. Indeed, most studies on interspecific comparisons of vocalizations have focused on quantitative aspects, such as the repertoire size (Catchpole, 1980; Kroodsma, 1977; MacComb and Semple, 2005). Whereas temporal aspects of vocal signals or vocal interactions have so far been little studied, they nonetheless could provide a wealth of information regarding the influence of social life on the evolution of vocal communication. We predicted that social life, in terms of the number of social partners or distance between partners for example, would affect the temporal structure of song.

We first estimated the proportion of discontinuous/continuous songs. Two categories of songs could be distinguished: *discontinuous songs*, corresponding to unitary notes or short motifs (a fixed combination of acoustic elements) produced at discrete intervals, and *continuous songs* in which long sequences are produced, with less than 0.5 s interval between two successive motifs.

For each species, we measured: 1- sequence duration, 2- intervals between two successive sequences or two successive discontinuous motifs, 3- the motif duration, 4- the number of motifs per sequence, 5- intervals between two successive motifs within a sequence.

#### Results

The four species showed clear differences in the temporal organization of their song. Considering the proportion of continuous and discontinuous songs, a gradient was observed from *O. morio*, that produced only single song elements (categorized as “whistles”) to *S. bicolor* that produced only long phrases of continuous song (categorized as “warbling”). *O. nabouroup* and *L. nitens* appeared intermediate, producing both categories of songs (Table 3).

**Figure 11:**
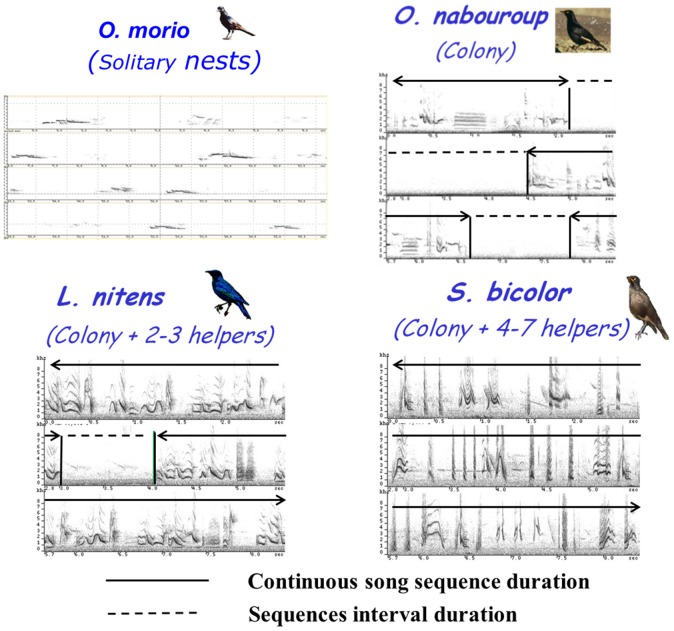
**Song sequence duration and sequence interval duration for the 4 species of African sturnids**.

Interestingly, this gradient corresponded to the increase in the complexity of social life (Figure 11): the more the species showed a complex and especially family type of social organization (in terms of number of congeners and nest proximity), the more their songs were produced in a continuous manner. In the same way, for species that produced continuous song, the phrase durations and the number of motifs per phrase increased following the same gradient (ANOVA, *F* = 5.51, *df* = 2, *p* < 0.0001; *F* = 89.82, *df* = 2, *p* < 0.0001 respectively).

On the other hand, the motif durations as well as the phrase intervals decreased following this “social” gradient (ANOVA, *F* = 11891, *df* = 2, *p* < 0.0001 *F* = 442, *df* = 2, *p* < 0.0001 respectively).

**Figure 12:**
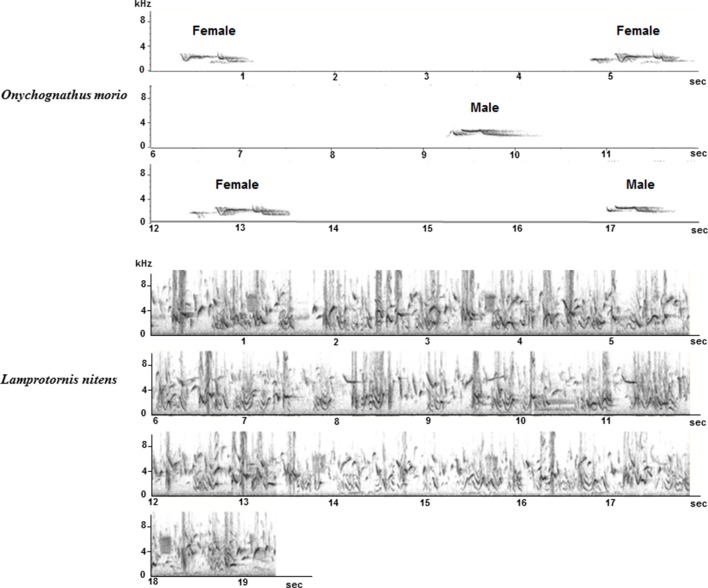
**Whistles of a male and a female ***O. morio*** (Top): whistles are separated by silent intervals**. Chorus of *L. nitens*: several birds are singing together and songs overlap.

Song overlap was never observed in *O. morio*. On the contrary, in *L. nitens* and *S. bicolor*, song overlap was very common and we frequently recorded choruses of birds living in the same group (Figure 12). Both alternating and overlapping song interactions are also regularly observed in *O. nabouroup*.

## Conclusion

The data presented here on one animal model reveal the interest of focusing on one question (here the temporal features of song that may or may not lead to alternating vocal interactions) and examining the different facets of the question. To the question: do European starlings show turn-taking in their vocal exchanges between males?, we can, from both observations and experiments, provide some answers: (1) they do favor alternation over overlapping, in particular through an immediate adaptation of the singer to the mere presence of another singing individual, but also according to the social situation and social density; (2) overlap does indeed disrupt the exchanges; (3) as in humans, there is an influence of context: alternation predominates in the usual interactions between males but chorusing can occur in more communal and intense social contexts (e.g., roosts), (4) the capacity for alternation develops during ontogenesis and social deprivation during development results in the inability to sing in a manner that favors “turn-taking” Social influences during development may directly affect the development of the brain processes devoted to song categorization.

While “turn-taking” is favored in the distant social interactions between males, more continuous song is produced in proximate interactions such as male-female interactions, or exchanges between close social partners (Hausberger et al., 1995). It has been proposed that warbling could play some stimulating role on the physiology of the listeners but also on the emitters too as found in budgerigars by Brockway (1969) and Adret-Hausberger and Jenkins (1988). Warbling is often associated with excitation behaviors such as visual displays and the production of high pitched trills, especially in the breeding season (Verheyen, 1980). As mentioned earlier, when producing warbling, male starlings seem to be “unaware” of the stimulation of their environment. Fundamentally, male starlings show movements of the head, typical of observation, during the silent interval between successive whistles and an erect posture while they are more in an oblique posture, with or without wing displays and a low reactivity while warbling.

In humans, it has been proposed that “attention is an intrinsic motivation for all utterances in a conversation, independent of the other possible motivation…” (Sacks et al., 1974). Excitation may lead to more overlap.

Interestingly, the comparative study of African starlings reflects these findings: the more communal the species, the more song overlap and choruses appear during close-range interactions, and the more continuous the song. The more territorial and long distant interactive a species is, the more alternation there is, hence the more discontinuous the song structures are. Some species like the European starling and the pale-winged starling show both song styles, reflecting the different contexts of interaction. Other species may also show this relationship between the temporal features of an interaction and the arousal states of the interactants: in barnacle geese *Branta leucopsis* triumph ceremonies, females that “encourage and support” their mate in the interaction will first alternate calling but with an increasing tempo and then overlap and chorus as excitation increases (Hausberger and Black, 1990) while those that do not support their partner (older pairs) produce other soft types of calls without any temporal synchronization (Bigot et al., 1995). According to Hauser (1992), the timing of calling in macaques may be altered in such a way that it is used by individuals to manipulate or facilitate social relationships.

One may speculate that the need for mutual intelligibility and information seeking but also the need for giving and receiving attention, a potential mediator of social bonding (Fedurek et al., 2013) may have constituted the basis for the evolution of turn-taking. Humans too may produce choirs that are perceived as a communal display rather than an interaction between individuals.

In the Dogons, as mentioned earlier, observing rules in language coincides with law and order in the society (Calame-Griaule, 1965). It is true too that spacing of the vocalizations requires calmness, control and attention toward the others instead of being self-centered. For France et al. (2001), the non-verbal cues that accompany turn-taking demonstrate mutual attention and responsiveness. According to Bourhis (1982) and Hofstede (1980), some human societies are built upon the development of “speaking well” while others, more communal, favor the knowledge of the social relationships. This is reminiscent of the gradient observed in species of the starling family (Sturnidae). Other communal breeders and group living animals such as the Australian magpies also favor choruses and overlap of songs (e.g., Brown and Farabaugh, 1997). At the other extreme, territorial skylarks have developed continuous songs that prevent turn-taking: the challenger deliberately overlaps the rival and “takes over” (Geberzahn and Aubin, 2014). This recalls some human conversations where the dominant individual disregards the other's turn.

For Takahashi et al. (2013a), vocal turn-taking does not require higher order cognitive capacities. Indeed the temporal features of animal vocal interactions in many ways parallel human communication. In particular, alternating vocal interactions are present in a large number of songbirds while cetaceans and primates seem to have “conversations” (e.g., Snowdon and Cleveland, 1984). However, as mentioned by Snowdon (1982), “in no way do they approach the complexity of human rules…they do indicate that rule-governed communication systems are not unique to humans. The use of rule systems for vocal communication is not limited to human beings.”

This review makes two additional points: turn-taking is one characteristic feature of human conversations but choruses might well be of interest if the social evolution of language and the intercultural aspects are to be considered; more integrative studies such as those described here (and in progress) for starlings are needed in order to tackle the question of the evolution of rule-governed communication in language.

### Conflict of interest statement

The authors declare that the research was conducted in the absence of any commercial or financial relationships that could be construed as a potential conflict of interest.
