# Corrigendum: Social coordination in animal vocal interactions. Is there any evidence of turn-taking? The starling as an animal model

**DOI:** 10.3389/fpsyg.2015.01924

**Published:** 2015-12-16

**Authors:** Laurence Henry, Adrian J. F. K. Craig, Alban Lemasson, Martine Hausberger

**Affiliations:** ^1^Laboratoire d'éthologie animale et humaine, Centre National de la Recherche Scientifique, UMR 6552, Université de Rennes 1Rennes, France; ^2^Department of Zoology and Entomology, Rhodes UniversityGrahamstown, South Africa; ^3^Laboratoire d'éthologie animale et humaine, Centre National de la Recherche Scientifique, UMR 6552, Station Biologique, Université de Rennes 1Paimpont, France

**Keywords:** turn-taking, vocal interactions, conversation rules, mammals, birdsong, sturnids

Figure 3 of the article by Henry et al. (2015) contained a minor error, which we correct here.

**Figure 3:**
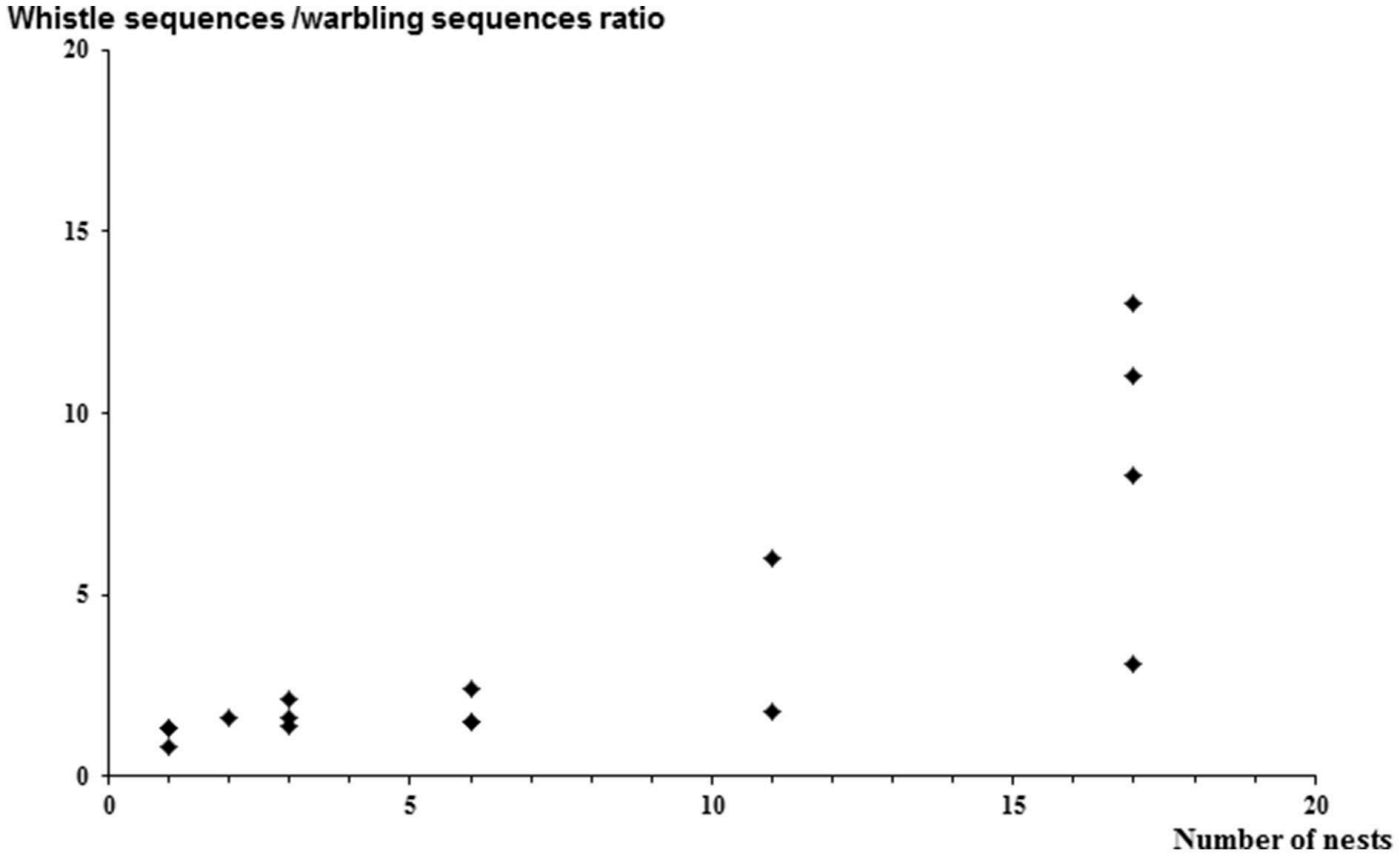
**Whistle sequences/warbling sequences ratio according to the number of nests per colony (***rs*** = 0.9, ***p*** = 0.003)**. Starlings produce more discontinuous song (whistles) when the number of neighbors is high (dense colonies).

Figure captions 4, 5, and 12 contained minor errors, which we correct here.

**Figure 4. Song style of birds belonging to colonies of different sizes**. Although the birds were recorded in very different conditions, a clear trend appeared toward an increase in whistling (hence discontinuous songs) and a decrease of warbling (hence continuous song) with increasing colony size (= number of neighbors). X: mean number of whistles per sequence (From Hausberger, 1997).

**Figure 5. Intervals separating two successive whistles produced by two different individuals during vocal interaction (overlap: when two whistles overlap)**. Most whistling exchanges show an interval of 2 s or less between the first and second whistle (arrow).

**Figure 12. Whistles of a male and a female *O. morio* (Top): whistles are separated by silent intervals. Choruses of *L. nitens***: several birds singing together with their songs in overlap.

## Conflict of interest statement

The authors declare that the research was conducted in the absence of any commercial or financial relationships that could be construed as a potential conflict of interest.

