# Supplementary material for: Social coordination in animal vocal interactions. Is there any evidence of turn-taking? The starling as an animal model
Source: Front Psychol. 2015 Sep 28;6:1416. doi: 10.3389/fpsyg.2015.01416 (PMC4585254; doi:10.3389/fpsyg.2015.01416)

Appendix 1: Song characteristics of 4 male adult captive starlings recorded in two situations: isolated in a cage without any contact with conspecifics or in a social group including several males and females. All birds increased the ratio whistles/warbling showing a modification of the singing style according to the context by producing a higher proportion of discontinuous song in the social context


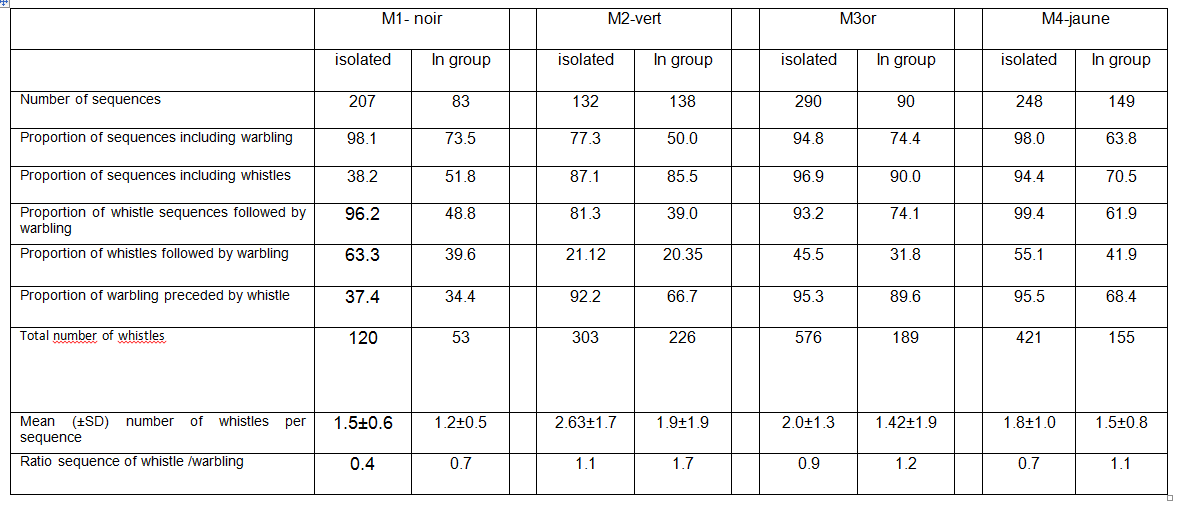

Supplement: Supplementary file 1 [file Table1.DOCX]
